# Sika Deer Carrying *Babesia* Parasites Closely Related to *B. divergens*, Japan

**DOI:** 10.3201/eid2008.130061

**Published:** 2014-08

**Authors:** Aya Zamoto-Niikura, Masayoshi Tsuji, Koichi Imaoka, Masanobu Kimura, Shigeru Morikawa, Patricia J. Holman, Haruyuki Hirata, Chiaki Ishihara

**Affiliations:** National Institute of Infectious Diseases, Tokyo, Japan (A. Zamoto-Niikura, K. Imaoka, M. Kimura, S. Morikawa);; Rakuno-Gakuen University, Hokkaido, Japan (A. Zamoto-Niikura, M. Tsuji, H. Hirata, C. Ishihara);; Texas A&M University, College Station, Texas, USA (P.J. Holman)

**Keywords:** Babesia divergens, sika deer, Cervus nippon, emerging disease, tickborne disease, zoonoses, Japan, parasites

**To the Editor:** Human babesiosis caused by *Babesia divergens*, a zoonotic pathogen of bovines in Europe, is an emerging tickborne disease (*1*). In the United States, a closely related *Babesia* sp. was identified in persons in Missouri and Kentucky and in eastern cottontail rabbits (*Sylvilagus floridanus*) on Nantucket Island, Massachusetts (*2*–*5*). We report that sika deer (*Cervus nippon*) in Japan also carry parasites genetically closely related to *B. divergens*.

During November 2007–February 2008 and November 2008–February 2009, we collected blood samples from 96 wild sika deer throughout Japan. We purified DNA from the blood, which had been stored in a freezer, by phenol/chloroform extraction and performed nested PCR for *B. divergens 18S rRNA* (*rDNA*), chaperonin-containing T-complex protein 1 eta subunit (*CCT7*, formerly described as CCTη) (*6*), and β-*tubulin* (*7*) genes. Primers for *rDNA* were designed from the sequences of related *Babesia* spp. (GenBank accession nos. U16370, U16369, and AY046575): dv101F (5′-ACAACAGTTATAGTTTCTTTGGTATTCG-3′) and dv1353R (5′-GCCTTAAACTTCCTTGCGGCTTAGAGC-3′), and dv159F (5′-GCTAATGCAAGTTCGAGGCCTTTTGGCG-3′) and dv1296R (5′-CGGACGAACCTTTTTACGGACACTAG-3′) for the first and second rounds, respectively. *CCT7* primers were similarly designed (GenBank accession nos. AB367924 and AB367925): Bdiv/odoCCTF1 (5′-CAAAATGAGYCACCTMCTCAACCTACC-3′) and BdivCCTR1 (5′-ATCTCAGCAGCTCACTACAGTGACCACCTC-3′), and Bdiv/odoCCTF2 (5′- CAACCTACCRATTCTCCTYYTGAAGGAGGG-3′) and BdivCCTR2 (5′-GGCTAATAAGTCGATATTGCGGGGCTCACG-3′) for the first and second rounds, respectively. The β*-tubulin* PCR protocol has been described (*7*).

Of the 96 blood samples, 12 from 5 prefectures (Hokkaido, Iwate, Tochigi, Nagano, and Miyazaki) were positive for *Babesia rDNA* (Technical Appendix). The sequence for sample 08–22 from Hokkaido (GenBank accession no. KC465978) was distinct from sequences of the other 11 samples (97.5%–97.6% identity in 1,041 bp), which consisted of 7 variant sequences (GenBank accession nos. KC465973–7 and AB857845–6) and 5 identical sequences (GenBank accession nos. KC465977 and AB861504–7) (99.7%–100% identity). The 5 identical sequences varied in only 1 of 909 bp from *B. divergens rDNA* from an *Ixodes persulcatus* tick in Russia [GenBank accession no. GU057385] (*8*).

β-*tubulin* (900 bp) was also amplified from the 12 *Babesia rDNA*–positive samples. Ten of the sequences consisted of 3 sequence variants (99.9% identity; GenBank accession nos. KC465969, KC465970, and KC465968/AB861508–14). The 2 divergent sequences (GenBank accession nos. KC465971 [08–22] and KC465989 [08–25]) were most similar to *B. odocoilei* (GenBank accession no. KC465972; 91% identity) and *Theileria orientalis* (GenBank accession no. AP011947; 79.9% identity), respectively. Thus, at least 1 deer likely had *Babesia* and *Theileria* spp. infections.

*CCT7* was amplified from 10 of the 12 *Babesia*-positive blood samples. The sequences (GenBank accession nos. KC465979–88) were more heterogeneous (98.7%–99.9% identity) than those for *rDNA* and β*-tubulin*; this finding was expected because *CCT7* evolves more quickly (*6*).

We generated a neighbor-joining phylogenetic tree (ClustalW, http://clustalw.ddbj.nig.ac.jp/index.php?lang=ja) from the *Babesia rDNA* sequences from our study and from GenBank (Figure, panel A). The tree shows a distinct lineage (Asian) cluster for the deer parasites, except for 08–22 (GenBank accession no. KC465978), within a clade also holding the *B. divergens* strains (human and bovine) from Europe (European Union lineage). The tree also shows a cluster encompassing *Babesia* spp. (human and rabbit) from the United States; *B. divergens* (deer), *B. capreoli* (deer), and *Babesia* sp. (chamois) from Europe; and *B. divergens* (human) from Portugal. Sequence 08–22 branches with *Babesia* spp. in *Ix. ovatus* ticks from Japan (GenBank accession nos. AY190123 and AY190124) (*9*). The branch lengths indicate clear separation between the isolates from sika deer and ticks, suggesting that the clustering may be attributable to the limited number of available related sequences.

**Figure:**
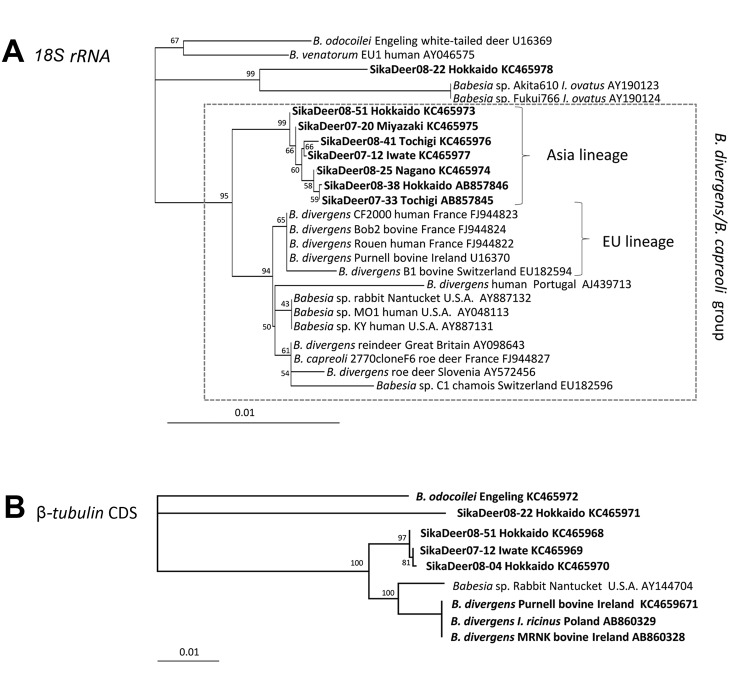
Neighbor-joining phylogenetic trees generated from *Babesia* sequences from GenBank and from our study in Japan, November 2007–February 2008 and November 2008–February 2009. Bootstrap support (1,000 repetitions) is indicated at the nodes. The trees are based on the partial (1,041 bp) *Babesia 18S rRNA* gene (A) and the partial (900 bp) β*-tubulin* gene (B). Sequences determined in this study are in boldface. Scale bar indicates the inferred number of substitutions per site. Lineages are indicated. CDS, coding DNA sequence.

We also generated a phylogenetic tree of β*-tubulin* sequences (900 bp), which produced similar topology and high bootstrap support (Figure, panel B). However, the limited number of relevant β*-tubulin* sequences precludes conclusions regarding the phylogeny of *Babesia* parasites. A *CCT7* phylogenetic tree was not generated because of the paucity of sequences. Overall, the phylogenies suggest that *B. divergens–* and *B. capreoli*–related parasites are found worldwide in temperate zones of the Northern Hemisphere, including Europe (*1*), the United States (*2*–*5*), Russia (*8*), and Japan.

We showed the presence of *B. divergens*–like *rDNA,* β*-tubulin*, and *CCT7* genes in sika deer from different Japanese prefectures, confirming the presence of this parasite in Japan. *B. capreoli*, which is serologically indistinguishable from *B. divergens*, was previously reported in sika deer (*10*). However, no molecular data for the *B. capreoli* isolate exist, so no conclusion may be drawn regarding its relationship to the *B. divergens*–like parasites from sika deer in our study.

There is an overabundance of wild sika deer in Japan because these animals easily adapt to a variety of climates, vegetation, and geography. Increased human exposure to deer habitats increases the risk of exposure to tickborne zoonoses, such as those caused by *Babesia* spp. In humans, infections caused by *B. divergens* and *B. divergens–*like parasites can be life threatening; fatality rates of 42% and 33% have been reported in infected asplenic patients in Europe and the United States, respectively (*1*–*3*). The findings from our study emphasize the need for increased clinical awareness of babesiosis in Japan and globally. They also emphasize the need for the swift diagnosis of suspected cases and prompt treatment of confirmed cases, especially in asplenic patients at high risk for the potentially deadly consequences of babesiosis.

Technical Appendix Map of Japan showing prefectures where *Babesia divergens*–like parasites were detected in sika deer.
