# Supplementary material for: Sika Deer Carrying Babesia Parasites Closely Related to B. divergens, Japan
Source: Emerg Infect Dis. 2014 Aug;20(8):1398–400. doi: 10.3201/eid2008.130061 (PMC4111200; doi:10.3201/eid2008.130061)
Supplement: Technical Appendix — Map of Japan showing prefectures where Babesia divergens–like parasites were detected in sika deer. [file 13-0061-Techapp-s1.pdf]

Article DOI: http://dx.doi.org/10.3208/eid2008.130061

Sika Deer Carrying *Babesia* Parasites Closely Related to *B. divergens*, Japan

Technical Appendix

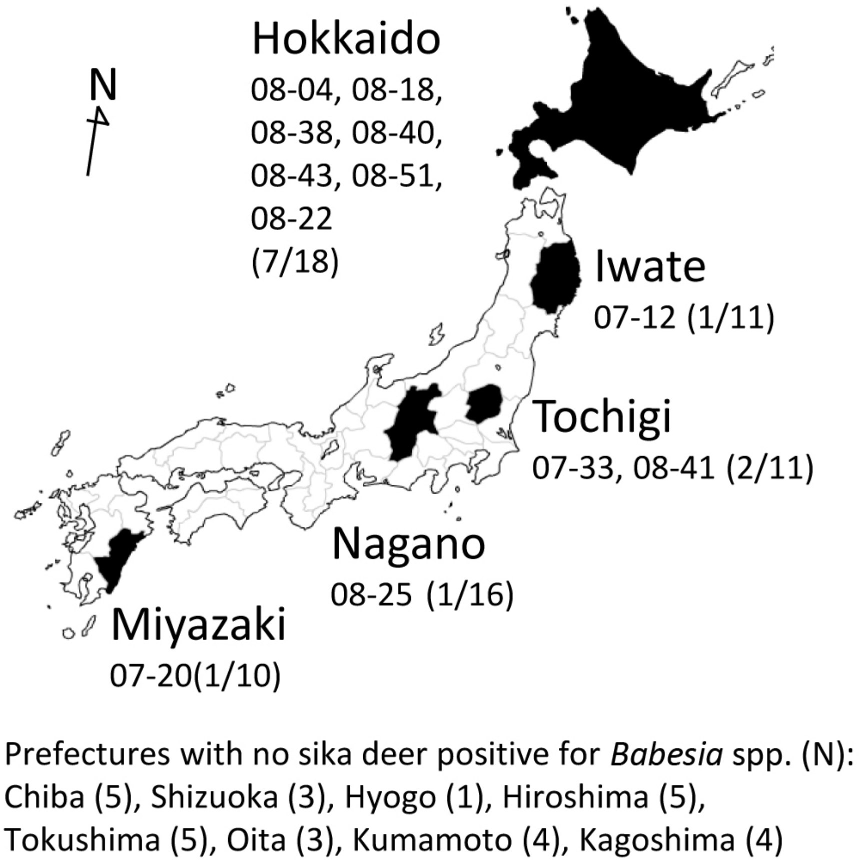

Technical Appendix Figure. Map of Japan showing prefectures where *rDNA* genes of *Babesia divergens*–like parasites were detected by PCR in blood samples from sika deer (black areas). Sample identification numbers are listed under prefecture names; numbers in parentheses indicate the number of positive samples/total number tested samples. A list of prefectures where no positive samples were found is shown at the bottom of the figure; the number of animals tested is shown in parentheses.
